# Biliatresone: progress in biliary atresia study

**DOI:** 10.1007/s12519-022-00619-0

**Published:** 2022-09-27

**Authors:** Jia-Jie Zhu, Yi-Fan Yang, Rui Dong, Shan Zheng

**Affiliations:** Department of Pediatric Surgery, Shanghai Key Laboratory of Birth Defect, and Key Laboratory of Neonatal Disease, Ministry of Health, Children’s Hospital of Fudan University, 399 Wan Yuan Road, Shanghai, 201102 China

**Keywords:** Biliary atresia, Biliatresone, Glutathione, Gut flora

## Abstract

**Background:**

Biliary atresia (BA) is one of the main causes of neonatal end-stage liver disease. Without timely diagnosis and treatment, most children with BA will develop irreversible liver fibrosis within the first two months. While current theorized causes of BA include viral infection, immune disorders, and genetic defects, the comprehensive etiology is still largely unknown. Recently, biliatresone attracted much interest for its ability to induce BA in both zebrafish and mice, so we summarized the latest progress of biliatresone research in BA and tried to answer the question of whether it could provide further clues to the etiology of human BA.

**Data sources:**

We conducted a PubMed search for any published articles related to the topic using search terms including “biliary atresia”, “biliatresone”, “GSH”, and “HSP90”. Relevant data were extracted from the original text or supplementary materials of the corresponding articles.

**Results:**

Biliatresone had shown its unique toxicity in multiple species such as zebrafish and mice, and pathogenic factors involved included glutathione (GSH), heat shock protein 90 (HSP90) and the related pathways. In combination with epidemiological evidence and recent studies on the intestinal flora in biliary atresia, a new pathogenic hypothesis that the occurrence of biliary atresia is partly due to biliatresone or its structure-like compounds depositing in human body via vegetables or/and the altered intestinal flora structure can be tentatively established.

**Conclusions:**

Based on the existing evidence, we emphasized that GSH and HSP90 are involved in the development of BA, and the maternal diet, especially higher vegetable intake of Asian women of childbearing age, accompanied by the altered intestinal flora structure, may contribute to the occurrence of biliary atresia and the higher incidence in the Asia group. However, the evidence from large sample epidemiological research is necessary.

**Supplementary Information:**

The online version contains supplementary material available at 10.1007/s12519-022-00619-0.

## Introduction

Biliary atresia (BA), characterized by fibro-inflammation and obstruction of extrahepatic bile ducts (EHBD) that can lead to liver fibrosis [1], remains the most common reason for liver transplantation in infants, and its clinical presentations include high levels of direct or conjugated serum bilirubin, acholic stool, dark urine, and progressive hepatic failure [2]. Typical pathophysiological features of BA livers include EHBD obstruction, portal tract fibrosis, ductular proliferation, and cholestasis with the appearance of bile plugs [3]. BA occurs variably worldwide, affecting all ethnic groups, and has a higher incidence in Asia and the Pacific region [4, 5]. To date, there are still no consistently curative treatment options for BA, and nearly 70% of children still cannot escape the fate of requiring liver transplantation even after undergoing a Kasai portoenterostomy (KPE), a procedure that removes fibrotic EHBD and reestablishes the bile drainage pathway [6]. Therefore, to transfer from effective but symptom-only treatments (such as KPE) to origin-related treatment and/or prevention methods, unraveling the etiology of BA must be a top priority.

Studies conducted on specimen samples (acquired during surgery or tissue biopsy) or experimental animal models, especially the Rhesus rotavirus (RRV)-induced mouse model, which replicates the key pathological features of human BA, such as EHBD damage, portal tract fibrosis and ductular proliferation [7], are trying to answer the question of what causes BA, and some of the factors have been uncovered. Since the theory of viral infection first proposed by Benjamin Landing in 1974, several viruses, including cytomegalovirus (CMV), have been reported to be related to the pathogenesis of BA [8, 9]; however, myxovirus resistance protein 1, the evidence of a preceding viral infection, could be found even in the absence of viral material, thus indicating that the virus infection detected may just be a coincidental prior event, and neither epidemiological investigations nor in vivo virological tests of BA patients confirm a certain virus that causes BA [10, 11]. Immune disorder is another possible cause that has been widely discussed. A variety of immune cells have been shown to be involved in BA [12]. A deficit in the number of circulating regulatory T cells (Tregs) in peripheral blood was reported [13], and the adoptive transfer of Tregs to pups before infection with RRV prevented the obstruction of EHBD [14], but subtle pathogenic mechanisms still need to be investigated. The identification of gene errors linked to BA remains elusive. A genome-wide association study carried out in a large cohort identified glypican-1 (*GPC1*) and adducin 3 (*ADD3*) as susceptibility factors of BA [15], and further research confirmed that abnormal localizations of *ADD3* and a *GPC1*-encoded protein exist in BA [16], but a more comprehensive, whole-genome mutation survey in a large cohort is required for further progress. While some pieces of confusion have been identified, the complete immunopathological mechanism of BA is still unclear. Recently, an environmental pathogenic factor, biliatresone, was found to induce BA in zebrafish and mice [17, 18]. Thus, we summarized the latest research progress on biliatresone and its toxicity-related pathogenic mechanisms, with the aim of providing new perspectives for future research on human BA etiology.

### Discovery

Biliatresone originated in livestock. The earliest case of neonatal death with jaundice in livestock was reported by Sven et al. in 1967. The autopsy suggested liver cirrhosis and cholestasis, but the cause was unknown [19]. In 1990, Harper et al. summarized the abnormal deaths in newborn flocks in New South Wales, Australia, in 1964 and 1988. After grazing on unconventional pastures affected by severe drought, the newborn flocks developed jaundice and died shortly after birth. Autopsy showed that the lambs had clay-colored stools in their intestines, with sclerotic liver and narrowed/disappeared EHBD. Further pathological examination revealed EHBD and liver fibrosis, cholestasis, and proliferation of intrahepatic cholangiocytes (IHCs), which were highly similar to human BA, but all ewes appeared normal [20]. The overlap of specific sites and similar pathologic findings suggested that the occurrence of BA in newborn lambs was related to some kind of environmental factor, but no further exploration was conducted by the author. Similar incidents occurred in subsequent years whenever pregnant ewes were exposed to specific pastures during the drought. Park et al. noticed the phenomenon in 2007. BA occurs in newborn lambs after long-term feeding of the *Dysphania* genus, which only appears in the dry season, suggesting that these plants may contain toxic substances that cause BA. Three crude fractions were then extracted with dichloromethane/methanol or water from the plants and verified in zebrafish larvae [21]. The results showed that the normal fluorescence intensity of the gallbladder of zebrafish exposed to the methanol fraction decreased significantly, indicating the presence of some biliary duct-specific toxins in the methanol fraction. After several rounds of separation and purification, the substance was successfully purified and named biliatresone (approximately 1.84% of the dry weight), which is also the first plant toxin known to selectively destroy the EHBD [22].

### Structure, chemical properties and synthesis

Koo et al. identified the molecular and structural formula of biliatresone with high-resolution mass spectrometry and nuclear magnetic resonance analysis in 2015. Biliatresone (molecular formula: C_18_H_16_O_6_) has a 1,2-diary-2-propenone structure, with an α-methylene ketone bridge formed between two phenyl groups; other functional groups include methoxy, hydroxyl and dioxyemethylene functional groups (Supplementary Fig. 1a) [23]. The chemical properties of biliatresone were later verified by Koo et al. with high-performance liquid chromatography and liquid chromatography‒mass spectrometry in 2016. The toxic core of biliatresone is the methylene relative to the α position of 1,2-diaryl-2-propen. The α-methylene, which functions as an electrophilic Michael receptor, undergoes Michael addition reactions with endogenous nucleophiles, including glutathione (GSH) and cysteine (D-NAC and L-NAC). Biliatresone can also react reversibly with established solvent adducts in vivo, including methanol adducts, water adducts, and hemiethylene cholate ketone (C_17_H_16_O_6_). Other functional groups, such as methylene dioxy, dimethoxy, and hydroxyl groups, also play important roles in the reaction [24]. Estrada et al. (USA) and Yang et al. (China) established an in vitro synthetic route of biliatresone in 2017 and 2019, respectively. The artificial product replicated the toxic effect of natural biliatresone and was verified through a zebrafish assay (Supplementary Fig. 1b, c) [25, 26].

**Fig. 1:**
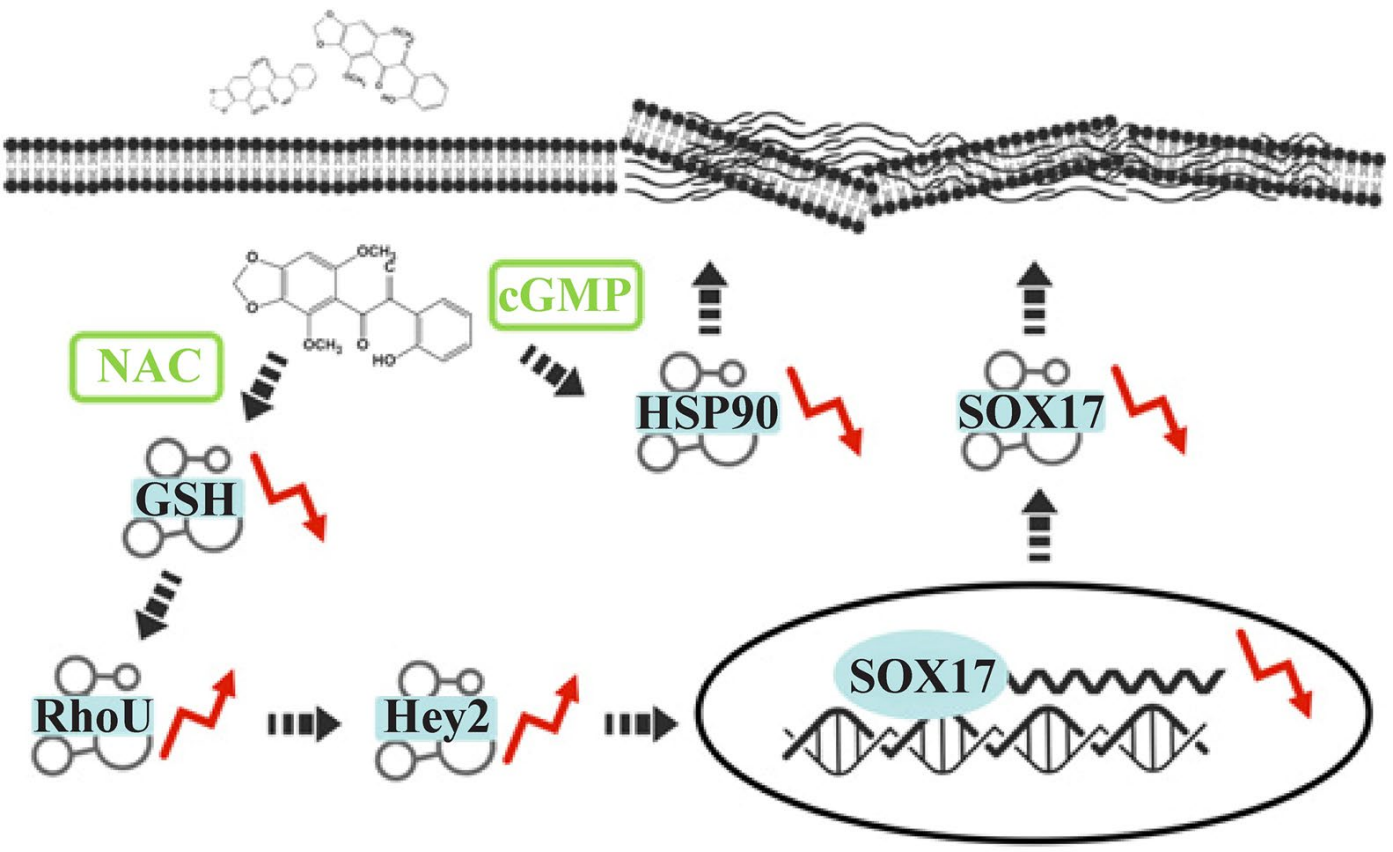
Mechanisms related to biliatresone-induced injury. *NAC* N-acetyl-L-cysteine, *cGMP* cyclic guanosine monophosphate, *HSP90* heat shock protein 90

### Biliatresone animal models

Animal models serve as an important tool for both etiological and damage mechanism studies of BA [27]. Although there is still a lack of direct evidence that biliatresone is the cause of human BA, the discovery of biliatresone does provide the possibility to establish animal models induced by environmental toxins, thus drawing a more complete map of the bile duct injury mechanism in BA. In 2015, Lorent et al. successfully established a biliatresone-induced zebrafish model with natural biliatresone. Experiments were carried out at different concentrations. The results showed that after 24 hours of treatment with biliatresone (0.5 μg/mL), all surviving larvae (93.3%, 14/15) had no significant changes in hepatocytes or intrahepatic bile ducts. Gallbladder showed varying degrees of shrinkage (3/14) or disappearance (11/14), and the effects were dose-dependent and time specific. Treatment on the 5th day post-fertilization (dpf) had more significant effects than intervention on the early stage (6 or 24 hours post-fertilization, hpf). Low-dose intervention (0.0625 μg/mL; 0.125 μg/mL) caused minor gallbladder injury, while the gallbladder underwent morphological changes (0.25 μg/mL) or severe deformation (0.5 μg/mL; 1.0 μg/mL) with increasing dosage, and the elimination of toxin (1.0 μg/mL) did not interrupt the progression of injury [22]. An artificial biliatresone-induced zebrafish model was established by Zhao et al. [28] and Yang et al. [29] in 2020. After treatment with biliatresone at 5 dpf for 24 hours (0.2–0.5 μg/mL) or 48 hours (0.15 μg/mL), the morphology of EHBD was destroyed, and the gallbladder disappeared in 12%–26% of larvae. Yang et al. subsequently constructed an artificial biliatresone-induced mammalian model with newborn BALB/c mice in the same year, and the study was conducted in both newborn and pregnant mice. The results showed that while 48.7% of newborn mice died within seven days after injection (24–48 hours after birth, 80 μg, intraperitoneal), jaundice, slower weight gain and bilirubinuria occurred in 40% of newborn mice within two to four days after injection. Pathological findings included integrity damage to the gallbladder and EHBD epithelium, liver fibrosis with inflammatory infiltration, IHCs hyperplasia in the portal area, and high expression of BA-specific diagnostic index matrix metalloproteinase 7 [29, 30]. All these features replicated the sight of human BA, suggesting it’s a good model for damage mechanism study of human BA (Table 1).

**Table 1:** Differences between Rhesus rotavirus-induced and biliatresone-induced biliary atresia models

Variables	RRV-induced mouse	Biliatresone-induced mouse	Biliatresone-induced zebrafish
Intervention	24–48 h after birth	24–48 h after birth	5 dpf
Dosage (time)	20 µL × 1.25–1.5 × 10^6^	80 µg	0.15 µg/mL (48 h) 0.2–0.5 µg/mL (24 h)
Jaundice	6–7 d post intervention	2–4 d post intervention	/
Clinical features	Jaundice, clay-like stool, growth retardation, bilirubinuria	Jaundice, clay-like stool, growth retardation, bilirubinuria	/
Success rate	75%	40%	12%-26% (artificial) 93.9% (natural)
MMP-7	Upregulation	Upregulation	/
Survival time	14 d	18 d	12 d
Biliary tract malformation	Existence	Existence	Existence
EHBD fibrosis	Existence	Existence	Existence
IHCs hyperplasia	Existence	Existence	Nonexistence

*RRV* Rhesus rotavirus, *MMP-7* matrix metalloproteinase-7, *EHBD* extrahepatic bile ducts, *IHCs* intrahepatic cholangiocytes, *dpf* days post-fertilization. "/" no data

### Damage mechanism

EHBD atresia is an important characteristic of BA. Lorent et al. further demonstrated the specific targeting of biliatresone to EHBD and gallbladder in 2015. Exposure to natural biliatresone (0.5 g/mL) caused loss of epithelial monolayer integrity in extrahepatic cholangiocytes (EHCs) and gallbladder epithelial cells, while hepatocytes showed only slight tubulin changes even with high doses (5 g/mL) of stimulation [22]. Inflammatory infiltration is another important feature of BA. Although inflammatory cell aggregation was observed in the portal vein region of the mouse model, the adaptive immune system of zebrafish was not established until 4–6 weeks after birth [31]. Lorent et al. showed that clearance of macrophages in zebrafish did not affect disease progression, indicating that differences exist in injury mechanisms among models, and the immune system plays a limited role in it [22, 29]. The above evidence suggests the following characteristics of biliatresone: specifically targeting EHBD and gallbladder, and perhaps not primarily dependent on the immune system.

### Toxicity to extrahepatic cholangiocytes depends on glutathione consumption

GSH plays a vital role in cellular protective antioxidant responses and has been shown to be involved in various cholestatic diseases, including BA [32–35]. Zhao et al. conducted RNA sequencing of biliatresone-treated zebrafish livers in 2016 and found that multiple genes related to GSH synthesis were upregulated after treatment, including *gclc*, *gclm* and *nrf2*. Mass spectrometry results showed that biliatresone caused a dose-dependent decrease in the level of GSH. The opposing fates of EHCs compared with hepatocytes during stimulation depend in large part on their differences in basal redox states. The GSH storage of EHCs was significantly lower than that of hepatocytes, and the reduction of GSH expression by BSO (a specific inhibitor of de novo glutathione synthesis) sensitized the EHCs to a low dose of biliatresone, while exogenous supplementation with NAC (precursor of GSH) alleviated the damage [36]. In 2020, Zhao et al. further revealed that in addition to differences in basal reserves, different parts of the liver had different ways to maintain GSH storage. Mutations in the glutathione reductase gene sensitize EHCs to low-dose biliatresone, while the same changes do not affect IHCs, which suggests that EHCs’s maintenance of GSH is more dependent on the conversion of GSSG (oxidized GSH) to GSH. IHCs may have a better synthetic ability to tolerate the toxin [28].

### GSH-RhoU-Hey2-SOX17 pathway

SOX17 is a master regulator of EHBD development in various vertebrate species, and reduced SOX17 expression induces hypoplastic gallbladders at late stages of organogenesis [37]. In 2016, Waisbourd-Zinman et al. found that biliatresone intervention resulted in decreased expression of GSH and SOX17 in mouse EHCs organoid [38], and the reduction in SOX17 expression by siRNA mimics the effect of biliatresone without affecting GSH levels [39]. In 2020, Sophia et al. applied gene chip technology and further found that the intracellular expression of RhoU/Wrch1 and Hey2 changed after treatment. RhoU/Wrch1 is a known regulator of cytoskeleton-related genes and actin organization, and the upregulation of RhoU disrupts epithelial cell polarization, which is necessary for tight junction assembly in MDCK cells [40]. Hey2 is a Notch signaling protein. The Notch signaling pathway is important for bile duct development regulation and plays an important role in various liver diseases [41]. The overexpression of RhoU/Wrch1 simulates the effect of biliatresone and simultaneously leads to an upregulation of Hey2 and downregulation of SOX17 without affecting the level of GSH, while overexpression of Hey2 can only decrease the expression of SOX17 without changing the expression of RhoU. This evidence jointly indicates that decreased GSH expression initiates a pathway leading to increased RhoU/Wrch1 expression, which in turn leads to a sequential increase in Hey2 expression and a decrease in SOX17 expression [42].

### The protein quality control mechanism

The heat shock protein 90 (HSP90) chaperone mechanism is a key regulator of protease homeostasis during eukaryotic cell physiology and stress conditions and is involved in many cellular processes, including protein folding and DNA repair [43, 44]. Rajagopalan et al. performed exome sequencing in 101 North American BA patients in 2020 [45]. The results suggested decreased HSP90 expression in BA and mutations in *STIP1* (a chaperone of HSP90) and *REV1* (a key role in DNA repair pathways) genes [45], which was consistent with the study of Dong et al. [46]. Zhao et al. reported evidence of the involvement of the protein quality control mechanism in biliatresone injury in 2020. The RNA sequencing results showed that heat shock response-related gene expression was upregulated after treatment with biliatresone and that the inhibition of HSP90 expression by the HSP90 inhibitor 17-N-allylamino-17-demethoxy-geldmycin made EHCs sensitive to a low dose of biliatresone. In addition, activation of cyclic guanosine monophosphate (cGMP) signaling rescued the damage caused by HSP90 decline, but NAC was unable to reverse the sensitization induced by HSP90 inhibition. This evidence suggests that the cGMP signaling pathway is independent of the GSH pathway and acts synergistically with NAC to reduce biliatresone-mediated damage by enhancing protein quality control [28]. A detailed map of the relevant mechanisms is shown in (Fig. 1).

### Environmental pathogenic factors of human biliary atresia

Biliatresone is a kind of 1,2-diarylacetone isoflavone that is rarely found in the environment. Currently, there is no evidence indicating that pregnant women could be directly exposed to biliatresone; however, Elliger et al. found in 1994 that beet roots inoculated with rhizocorrhiza produced an isoflavone with a similar biliatresone-like structure but without the necessary disease-causing core α-methylene [47, 48]. In 2002, Hur et al. screened the bacteria involved in phytoestrogen transformation in the human intestinal tract by 16S ribosomal RNA gene sequencing and found that clostridia existing in the human intestinal tract could split the C ring in the soybean isoflavone structure in vitro, resulting in the same methylate structure as the pathogenic structure of biliatresone [49]. These findings suggest that the human gut microflora has the ability to alter the structure of isoflavone in plants and that nontoxic precursors in food are likely to be converted to toxic substances through the gut microflora. The intestinal flora has been widely reported to be associated with liver functions in various liver diseases [50, 51]. Recently, the role of intestinal flora in BA has received attention. A disturbed gut microbiota structure was identified in the BA group [52] and correlated with the occurrence of postoperative cholangitis and jaundice clearance [53, 54]. Wang et al. conducted 16S ribosomal RNA gene sequencing on the intestinal flora of 16 BA patients after surgery in 2020, and the results showed that the intestinal flora diversity of BA patients decreased and the overall structure changed, among which the proportion of *Clostridium* decreased significantly [55]. Jee et al. found in 2022 that modification of intestinal microbiota composition by butyrate intake in newborn mice significantly improved EHBD injury induced by RRV, and this effect was associated with increased fecal *Clostridium* [56]. However, it is unknown whether this change in intestinal flora structure facilitates the transformation of nontoxic precursors in food.

BA occurs worldwide; the epidemiological survey showed that the worldwide distribution of BA was ethnically influenced. The incidence of BA is higher in Asia and the Pacific, among which the prevalence rate in the Chinese population (1:5000) [57] is approximately four times higher than that in North America (1:20,000) [58]. Regarding diet structure, a global diet analysis published in Lancet in 2019 showed that while the daily vegetable intake in both East Asia and the Pacific is above the global average, that in America is less than half the level [59]. A more detailed diet report summarized by Pomerleau et al. in 2005 showed that the daily fruit and vegetable consumption of Chinese women (15–44 years old) (325.5 g/day) was approximately 1.3 times higher than that of North American women (15–44 years old) (247.5 g/day) [60]. In addition, a study to identify potential risk factors for isolated BA conducted by The et al. in 2007 indicated that low maternal intake of some specific nutrients, including vitamin E, copper, phosphorus, and beta tocopherol, was associated with the occurrence of isolated BA [61]. Although the evidence is still limited, these results, including the discovery of biliatresone, remind us that diet structure could be one of the possible triggers of BA. Combined with epidemiological data, we try to establish such a hypothesis that food diet serves as a potential environmental factor of BA. The structural analogs in some specific foods are enriched in the intestinal tract of the Asian population and then transformed into toxins similar to biliatresone through the altered intestinal flora in BA. These toxins eventually affect the normal biliary system development of the fetus or newborn through the placenta or breast milk and ultimately lead to the occurrence of BA alone or accompanied by other environmental factors, such as CMV infection, which was verified to exist in 48%–50% of Chinese BA patients and is much higher than that of the European group (approximately 10%) [62]. In recent years, BA has been hypothesized to have begun as early as in utero, and syndromic BA is presumed to be an embryonic defect due to its unique visceral anomalies, such as polysplenia or situs inversus, although the actual genes involved are inconclusive [63]. Approximately 22.2% of fetuses diagnosed with hilar hepatic cysts develop cystic BA after birth, but it is not usually accompanied by organ abnormalities other than liver and gall [64, 65]. As for isolated BA, the evidence shows that it may develop in utero from the increased blood direct bilirubin levels that can be detected in the first hour after birth [1, 66]. These studies provide some extra support for our hypothesis, but larger cohort studies are needed for evidence based on epidemiology.

## Conclusions

Biliatresone is a kind of toxin that specifically targets EHCs and the gallbladder. GSH serves as a key molecule responsible for the toxic effects. Differences in the storage, synthesis and regeneration of GSH in various parts of the liver are the basis of injury, and the HSP90 pathway is also involved, but the mechanism has not been confirmed in mammalian models other than zebrafish. The discovery of biliatresone suggests that exposure to structure-related compounds from food or other environmental sources may be an important trigger event for BA in humans, and it is speculated that diet, especially the high vegetable intake in Asian populations, may be one of the reasons for the high incidence accompanied by the altered intestinal flora structure.

Future studies should focus on (1) the enrichment of biliatresone and structural analogs in the human environment, their relationship with the spatial distribution characteristics of BA cases and whether the corresponding structural analogs have the same toxicological effects in vivo; (2) the role of intestinal flora in BA development, especially the difference in intestinal flora structure between Asia and Western BA groups; and (3) the important roles of redox homeostasis and protein quality control mechanisms in BA in animal models other than zebrafish.

## Supplementary Information

Below is the link to the electronic supplementary material.

Supplementary file 1 (PDF 135 KB)

## Data Availability

All data generated or analyzed during this study are included in this published article and its supplementary information files.
