# Supplementary material for: Biliatresone: progress in biliary atresia study
Source: World J Pediatr. 2022 Sep 27;19(5):417–24. doi: 10.1007/s12519-022-00619-0 (PMC10149470; doi:10.1007/s12519-022-00619-0)
Supplement: Supplementary file 1 — (PDF 135 KB) [file 12519_2022_619_MOESM1_ESM.pdf]

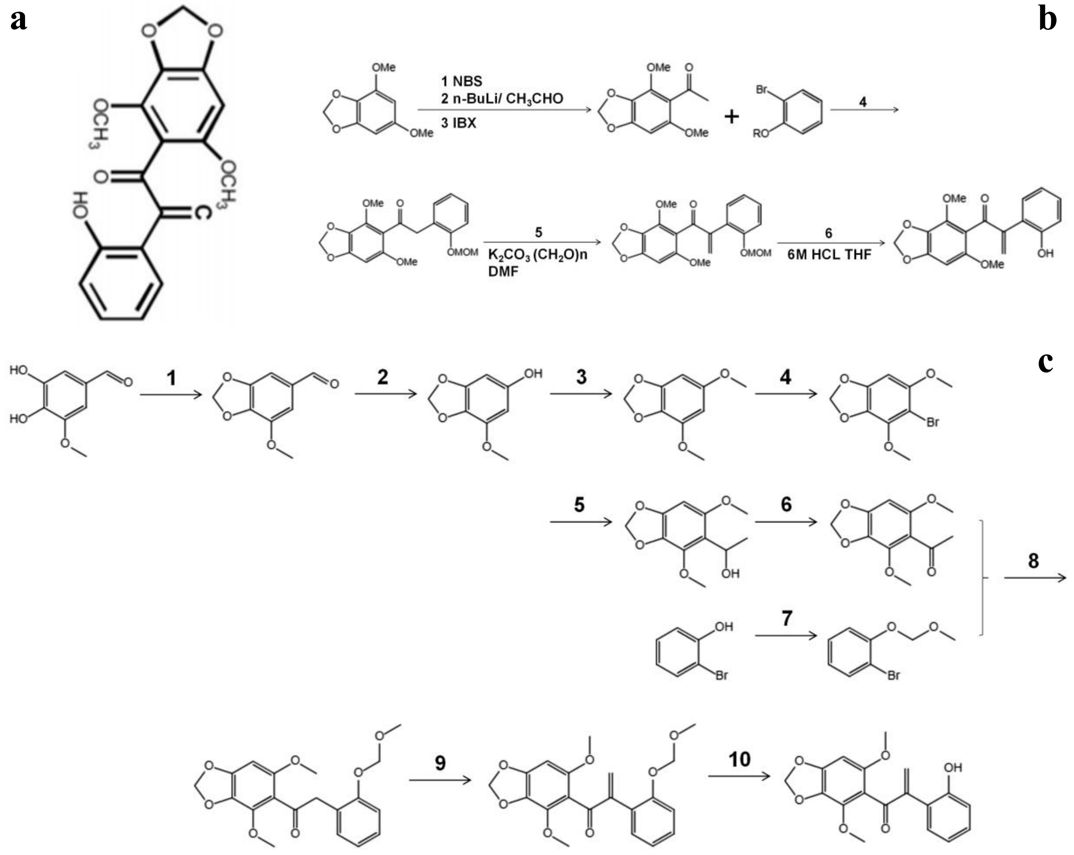

Supplementary Fig. 1 Structural formula and in vitro synthetic route of biliatresone. **a** Structural formula of biliatresone; **b** in vitro synthetic route of biliatresone (Yang et al. [26]); **c** in vitro synthetic route of biliatresone (Estrada et al. [25])
